# Chiral Effect at Nano-Bio Interface: A Model of Chiral Gold Nanoparticle on Amylin Fibrillation

**DOI:** 10.3390/nano9030412

**Published:** 2019-03-11

**Authors:** Jing Li, Rui Chen, Shasha Zhang, Zhongjie Ma, Zhuoying Luo, Guanbin Gao

**Affiliations:** 1 State Key Laboratory of Advanced Technology for Materials Synthesis and Processing, Wuhan University of Technology, No.122 Luoshi Road, Wuhan 430070, China; jingli@whut.edu.cn (J.L.); chenrui514@whut.edu.cn (R.C.); zjsymzj@whut.edu.cn (Z.M.); luozhuoying@whut.edu.cn (Z.L.); 2 School of Arts and Media, Wuhan Vocational College of Software and Engineering, No. 117 Guanggu Road, Wuhan 430205, China; shashazhang1987@139.com

**Keywords:** chiral AuNPs, nano-bio interface, amylin, fibrillation

## Abstract

Protein/Peptide amyloidosis is the main cause of several diseases, such as neurodegenerative diseases. It has been widely acknowledged that the unnatural fibrillation of protein/peptides in vivo is significantly affected by the physical and chemical properties of multiscale biological membranes. For example, previous studies have proved that molecule chirality could greatly influence the misfolding, fibrillation and assembly of β-Amyloid peptides at the flat liquid-solid surface. However, how the nanoscale chirality influences this process remains unclear. Here we used gold nanoparticles (AuNPs, d = 4 ± 1 nm)—modified with *N*-isobutyl-*L*(*D*)-cysteine (*L*(*D*)-NIBC) enantiomers—as a model to illustrate the chiral effect on the amylin fibrillation at nano-bio interface. We reported that both two chiral AuNPs could inhibit amylin fibrillation in a dosage-dependent manner but the inhibitory effect of *L*-NIBC-AuNPs was more effective than that of *D*-NIBC-AuNPs. In-situ real time circular dichroism (CD) spectra showed that *L*-NIBC-AuNPs could inhibit the conformation transition process of amylin from random coils to α-helix, while *D*-NIBC-AuNPs could only delay but not prevent the formation of α-helix; however, they could inhibit the further conformation transition process of amylin from α-helix to β-sheet. These results not only provide interesting insight for reconsidering the mechanism of peptides amyloidosis at the chiral interfaces provided by biological nanostructures in vivo but also would help us design therapeutic inhibitors for anti-amyloidosis targeting diverse neurodegenerative diseases.

## 1. Introduction

Protein/peptides amyloidosis is tightly associated with a variety of causes of diseases [1,2], such as neurodegenerative diseases [3,4,5,6]. It has been pointed out that the critical concentration of all these proteins/peptides fibrillation in vitro are far larger than their native concentration in vivo by several orders of magnitude [7]. More and more evidences have indicated that the multiscale biological membranes in vivo provide a larger number of reactive sites to interact with dissociative protein/peptides [8,9,10,11], which play a key procedure for their accumulation and fibrillation at very low concentrations [12,13,14]. Various models with different physicochemical properties (e.g., composition [15,16], surface charge [17], hydrophilicity [18], hydrophobicity [19,20], chirality [21,22,23,24] and size [25,26]) of interfaces have been built to simulate the interaction between peptides and membranes [27]. Some of our previous works [21,28] have verified the chiral effect on Aβ peptides fibrillation and assembly on flat solid-liquid interface. We also have reported the size effect of interfaces on Aβ peptides aggregation and fibrillation [26]. These inspired us to introduce the molecule chirality onto the nano-bio interfaces to study the chiral effect on protein amyloidosis.

As a kind of emerging nanoparticles, besides other advantages like high specific area, satisfactory water solubility [29,30], high quantum-yield [31,32], biocompatibility [33] and excellent stability, chemically modified gold nanoparticles (AuNPs), especially biomolecule modified AuNPs [34], also possess a distinct ultra-small nano-bio interface similar to ultra-small biomembranes, which suggests that AuNPs could act as an ideal model to investigate the chiral effect at nano-bio interface on amylin fibrillation when their surface are modified with chiral molecules.

**Scheme 1:**
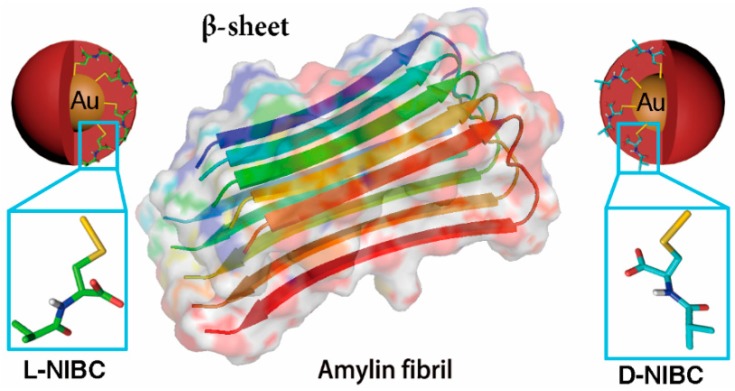
Gold nanoparticles (AuNPs) modified with *L(D)*-NIBC can serve as an ideal platform to study the chiral effect on amylin amyloidosis at ultra-small nano-bio interface.

For this purpose, we developed a pair of chiral AuNPs through chemical modification with *L*(*D*)-NIBC to study amylin amyloidosis on ultra-small nano-bio interface (Scheme 1). We discovered that both chiral AuNPs (both average size: 4 ± 1 nm) could inhibit amylin aggregation and fibrillation in a dosage-dependent manner. Thioflavine-T (ThT) assay showed that chiral AuNPs played inhibitory roles in the nucleation phase. Atomic force microscopy (AFM) images showed there were no obvious fibril formation intuitively when the addition of chiral AuNPs over 20 mg·L^−1^, while the amorphous structures or short rod-like aggregates appeared in the corresponding samples with addition of *L*- or *D*-NIBC-AuNPs. Dynamic light scattering (DLS) results showed that chiral AuNPs could prevent amylin forming oligomers. In-situ real time CD spectra showed that *L*-NIBC-AuNPs could inhibit the conformation transition process of amylin from random coils to α-helix, while *D*-NIBC-AuNPs could only delay but not prevent the formation of α-helix, however they could inhibit the further conformation transition process of amylin from α-helix to β-sheet. These results not only provide interesting insight to reconsider the mechanism for peptides amyloidosis at the chiral interfaces provide by biological nanostructures in vivo but also would help us design therapeutic drugs for diverse neurodegenerative diseases.

## 2. Materials and Methods

### 2.1. Reagents and Materials

Amylin (1.0 mg package, lot number: 1556842) was purchased from AnaSpec Inc. (San Jose, CA, USA) 1,1,1,3,3,3-Hexafluoro-2-propanol (HFIP, 99.8% ACS reagent grade), *N*-isobutyl-*L*(*D*)-cysteine (*L*(*D*)-NIBC, 99.9%, Bio reagent grade), ThT was purchased from Sigma-Aldrich (St. Louis, MO, USA). Hydrogen tetrachloroaurate (III) hydrate (HAuCl_4_·3H_2_O, 99.999%) was purchased from Acros Organics (Brussels, Belgium). Sodium borohydride (NaBH_4_, >96%) was purchased from Sinopharm Chemical Reagent Co., Ltd. (Shanghai, China). Phosphate buffered saline (PBS) plates were purchased from Sangon Biotech (Shanghai, China). The regenerated cellulose dialysis membranes with a molecular weight cut off (Mwco) of 2500D was purchased from Yobios BioTech (Xi’an, China). The ultrafiltration device (Mwco: 30kD) was purchased from Merck Millipore (Darmstadt, Germany). All glass vessels were soaked with aqua regia (volume ratio = 3:1, HCl/HNO_3_) for 12 h, washed with ultrapure water several times, being dried out, then soaked in chromic acid lotion for 30 min and thoroughly washed by ultrapure water, dried in an oven before use.

### 2.2. The Preparation of L(D)-NIBC-AuNPs

The synthesis of *L*(*D*)-NIBC-AuNPs was referred to in Reference [26]. In brief, *L*(*D*)-NIBC (3.2 mg) was introduced to the mixture of HAuCl_4_ (0.2 mL, 25 mmol·L^−1^, in methanol) and acetic acid (0.05 mL) under vigorous stirring in the ice bath. After the orange solution turned cloudy, 0.1 mL NaBH_4_ (0.1 mmol·L^−1^) was immediately added and stirred for 1 h with an ice bath. The resulting mixture was terminated by adding moderate acetone to obtain precipitate. The raw products were dialyzed in water for 7 days to remove excess reactant, organic solvents and small-sized gold nanoparticles. Then, the products were moved into an ultrafiltration device for centrifugation separates (Rcf: 5000 g, 40 min). The solution in the centrifuge tube was discarded to remove the large-sized gold nanoparticles and the solution in the filter device and water was added to the centrifuge tube for centrifugation separates, repeated for three times. Then the solution in the filter device was collected and lyophilized at a low temperature. The lyophilized powders were stored at 4 °C for the following experiments.

### 2.3. Sample Preparation of Amylin Solution

To remove pre-existing aggregation structures, according to Reference [35], amylin powder was dissolved in hexafluoro-2-propanol (HFIP) at 1 mg·mL^−1^ in a sealed vial. Then the solution was sonicated for 2 h in the ice bath. After removal of the solvent with a gentle stream of N_2_ gas, dried amylin powder was re-dissolved in 200 μL HFIP and divided into several vessels, stored in −20 °C for future use. Before the experiment, the amylin/HFIP solution was dried out by N_2_ gas and was dissolved in 10 mmol·L^−1^ PBS (pH = 7.4) at a final concentration of 25 μmol·L^−1^. The freshly prepared amylin solutions were used in all the following experiments.

### 2.4. UV-Vis Spectra Measurement

UV-Vis spectra were performed on a Shimadzu UV-1800 UV-Vis spectrophotometer in the range of 300 to 1000 nm at a scan rate of 0.5 nm·s^−1^. The lyophilized *L*(*D*)-NIBC-AuNPs were dissolved in the water with a final concentration of 1000 mg·L^−1^ and then put it into the high-quality quartz cell for testing.

### 2.5. Infrared (IR) Spectra Measurement

Infrared (IR) spectra were performed on a Bruker Vertex 80v Fourier transform infrared (FT-IR, Karlsruhe, Germany) spectrometer in the range of 4000–400 cm^−1^ with 64 scan times. The lyophilized *L*(*D*)-NIBC and *L*(*D*)-NIBC-AuNPs were tested by attenuated total reflectance (ATR) mode in a vacuum atmosphere (vacuum degree < 5 Pa) at room temperature.

### 2.6. Dynamic Light Scattering (DLS) Measurement

Dynamic light scattering (DLS) measurements were performed on a Malvern Nano-ZS ZEN3600 zetasizer (Malvern Panalytical, Malvern, UK). The lyophilized *L*(*D*)-NIBC-AuNPs were dissolved in the water with a final concentration of 50 mg·L^−1^ (volume: 1.5 mL) and then put it into the high-quality quartz cell for testing at 37 °C for 5 times in general mode. To monitor the time-dependent diameters or time-dependent zeta potential of amylin aggregates in presence of *L*(*D*)-NIBC-AuNPs, 1.5 mL solution containing 10 μmol·L^−1^ amylin with the absence or presence of 20 mg·L^−1^ *L*(*D*)-NIBC-AuNPs were added into a high-quality quartz cell (for size) or a DTS1070 folded capillary cell (for Zeta) and then the samples were tested with protein mode continuously for 24 h at 37 °C.

### 2.7. Transmission Electron Microscopy (TEM) Measurements

Transmission electron microscopy (TEM) measurements were performed on JEOL 200CX TEM (JEOL Ltd., Tokyo, Japan) with the accelerating voltage of 200 kV. For sample preparation, the lyophilized *L*(*D*)-NIBC-AuNPs were dissolved in 80% (volume ratio) aqueous alcohol and 10 μL solutions were dropped onto the 200 mesh carbon-coated copper grid, leaving it dried out.

### 2.8. Thioflavine-T (ThT) Binding Assay

ThT binding assays [36] were performed in a 96-well plate with a total volume of 250 μL. ThT solution (20 μmol·L^−1^) was prepared freshly in deionized water at pH 7.4. Freshly dissolved amylin solution was prepared at a concentration of 125 μmol·L^−1^ in phosphate buffer (20 mmol·L^−1^, pH = 7.4) and diluted to final concentrations of 50, 20, 10, 5 and 2 μmol·L^−1^ with PBS (10 μmol·L^−1^, pH = 7.4). Then, 50 μL of ThT stock solution (20 μmol·L^−1^) was added to the peptide solution. Fluorescence kinetics were measured on a Synergy^TM^ MX MultiMode Microplate Reader, with a time interval of 10 min at 37 °C. The excitation wavelength of ThT fluorescence was 445 nm and the emission wavelength was 485 nm. All experiments were repeated 3 times.

### 2.9. Circular Dichroism (CD) Spectra Measurement

Circular dichroism (CD) spectra measurements were performed on Jasco-1500 CD spectrometer. The lyophilized *L*(*D*)-NIBC-AuNPs were dissolved in water with a final concentration of 50 mg·L^−1^ (volume: 3 mL). Besides, the preformed fibrils were prepared by incubating amylin in the absence or presence of 20 mg·L^−1^ *L*(*D*)-NIBC-AuNPs for 24 h at 37 °C. The samples were loaded in a quartz cell with an optical path length of 0.1 cm. The scanning speed was 50 nm·min^−1^, the data pitch was 0.1 nm, the response time was 4 s and the band width was 1 nm. Raw data were processed by subtraction of buffer spectra followed by smoothing, in accordance with the manufacturer’s instructions.

### 2.10. Atomic Force Microscopy (AFM) Characterization

The morphology of amylin aggregates was characterized by atomic force microscopy (AFM). Samples were dropped onto freshly cleaved mica surface and then dried by N_2_. The AFM experiments were performed on a FastScan (Bruker) with tapping mode. All images were recorded at the 512 × 512-pixel resolution at scan rate of 1.0 Hz with FastScan-Air tips.

### 2.11. Cell Experiment

Rat insulinoma cells (INS-1 cell) were cultured in RPMI 1640 with 10% (*v*/*v*) fetal bovine serum (FBS, Gibco) and 1% penicillin/streptomycin in a 95% humidified incubator (Thermo) containing 5% CO_2_ at 37 °C. INS-1 cells were incubated in a 96-well plate at 37 °C with the density of 10,000 cells per well in 200 μL of culture medium for 24 h. Next, the same amount of *L*(*D*)-NIBC-AuNPs were mixed with INS-1cells culture medium with the finally concentration of 50, 20, 10, 5, 1 mg·L^−1^. The cell viabilities were evaluated by CCK-8 assay. Moreover, the ultra-pure water and the *L*(*D*)-NIBC enantiomers were used as the blank and the relevant controls to explore the cytotoxicity of *L*(*D*)-NIBC modified AuNPs. The pipette tips and test tube were sterilized with high temperature steam. The operating table was sterilized under the ultraviolet light for at least 8 h before experiment. The operation carried out on the operating table under the flame of an alcohol lamp.

## 3. Results and Discussion

### 3.1. The Characterization of L(D)-NIBC-AuNPs

**Figure 1:**
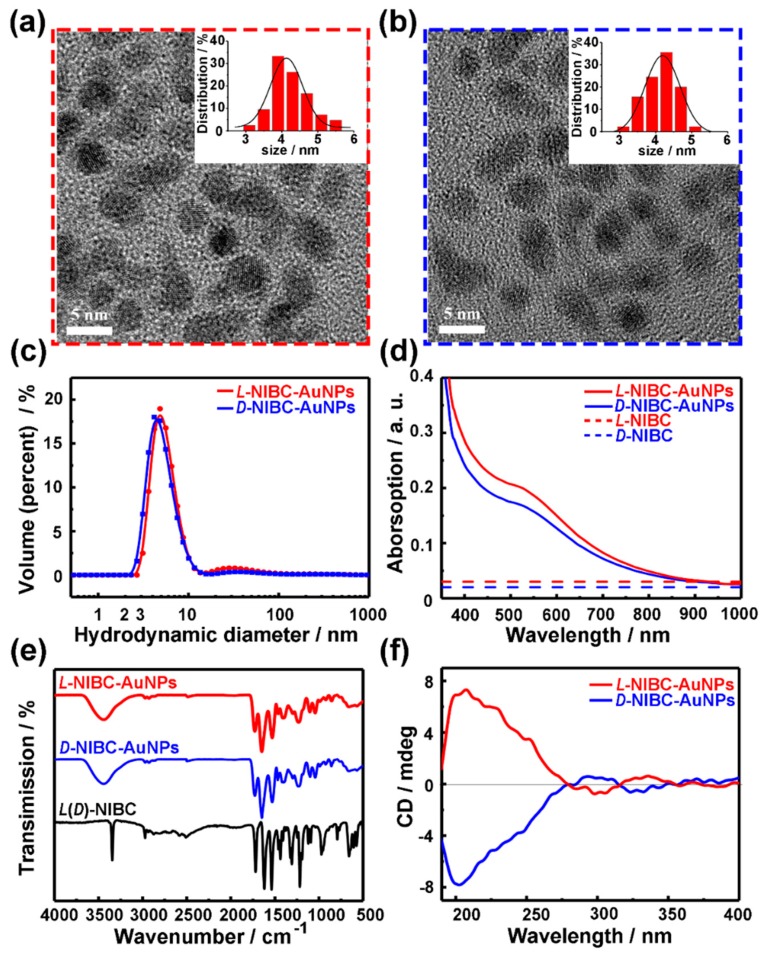
HR-TEM images of *L*-NIBC-AuNPs (**a**) and *D*-NIBC-AuNPs (**b**). Insets of a and b are the histograms of diameter distribution for *L*(*D*)-NIBC-AuNPs. DLS results (**c**), UV-Vis spectra (**d**), FT-IR spectra (**e**) and CD spectra (**f**) of *L*-NIBC-AuNPs (red curve) and *D*-NIBC-AuNPs (blue curve). Dotted lines in (**d**) and black curve in (**e**) represent *L*(*D*)-NIBC.

L(D)-NIBC-AuNPs were synthesized according to Reference [26]. Figure 1a,b and Figure S1 in the Supplementary Materials showed the high resolution TEM images of L(D)-NIBC-AuNPs, both of them presented nearly spherical. The insets of Figure 1a,b showed the histograms of diameter probability distribution for chiral AuNPs based on the corresponding high resolution images taken by TEM. The statistical data indicated that their average diameters of both *L*-NIBC-AuNPs and *D*-NIBC-AuNPs were 4.0 ± 1.0 nm. The hydrodynamic diameter distribution of both *L*-NIBC-AuNPs and *D*-NIBC-AuNPs in ultra-pure water measured by the DLS were 6 ± 2 nm (Figure 1c), which were slightly larger than the corresponding statistical diameters measured by the TEM. The UV-Vis spectra of *L*(*D*)-NIBC-AuNPs were given in Figure 1d. The typical spectroscopic absorption feature of *L*(*D*)-NIBC-AuNPs appeared the shoulder peaks around 520 nm, which were in accordance with the surface plasmon resonance of AuNPs of 5 nm [37,38,39]. Figure 1e showed the FT-IR spectra for *L*(*D*)-NIBC and *L*(*D*)-NIBC-AuNPs. The disappearance of –S–H stretching vibration absorption (around 2520 cm^−1^) in FT-IR spectra of *L*(*D*)-NIBC-AuNPs (Figure 1e) indicated that *L*(*D*)-NIBC molecules were successfully grafted onto AuNPs via Au-S bond. In addition, the curve of *L*-NIBC-AuNPs was corresponding to that of *D*-NIBC-AuNPs in the IR spectra (Figure 1e), which implied that the functionalization degree of two chiral AuNPs was similar. The CD spectra of chiral AuNPs (Figure 1f) presented corresponding mirror images between *L*-NIBC-AuNPs and *D*-NIBC-AuNPs. The XPS spectra showed that the oxidation states of Au in the surface of both two chiral AuNPs were between that of Au(I)−thiolate complexes and large Au(0) nanocrystals (Figure S2 in the Supplementary Materials), which was consistent with the typical characteristics of AuNPs. These results indicated that the chiral enantiomers were grafted onto the surface of AuNPs. Namely, the chiral nano-bio interfaces were constructed successfully.

### 3.2. L(D)-NIBC-AuNPs on the Fibrillation of Amylin

**Figure 2:**
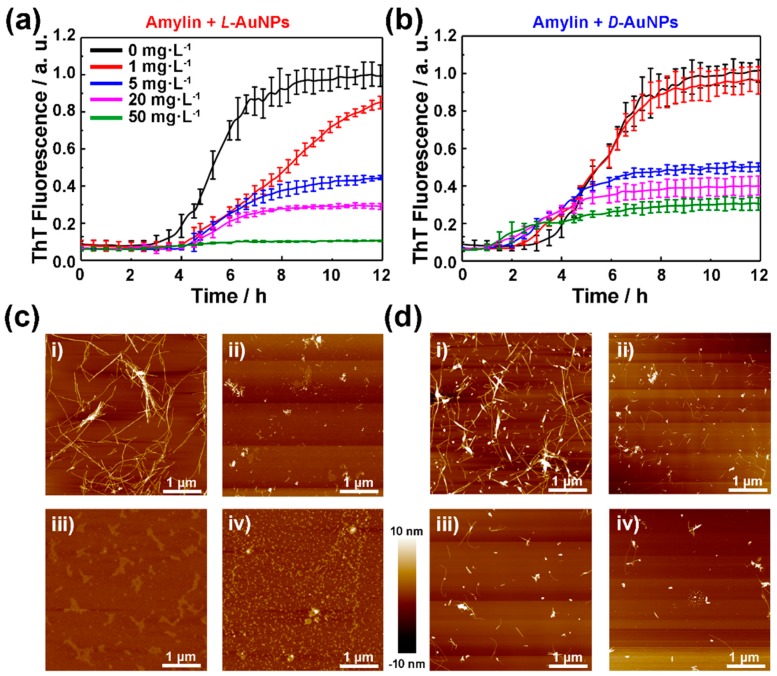
Time-dependent ThT fluorescence profiles for 10 μmol·L^−1^ amylin in PBS (pH 7.4) with the presence of *L*-NIBC-AuNPs (**a**) or *D*-NIBC-AuNPs (**b**) with different concentrations at 37 °C. The colors of the curves represented different concentrations of *L*(*D*)-NIBC-AuNPs, black: 0, red: 1 mg·L^−1^, blue: 5 mg·L^−1^, purple: 20 mg·L^−1^, green: 50 mg·L^−1^; each curve displayed the average data of three parallel experiments. Data points and error bars shown represent mean ± SE, (*n* = 3). AFM images (**c**,**d**) of the samples collected at the end of the ThT assays with different concentrations of 1 mg·L^−1^ (**i**), 5 mg·L^−1^ (**ii**), 20 mg·L^−1^ (**iii**) and 50 mg·L^−1^ (**iv**) of *L*-NIBC-AuNPs (**c**) and *D*-NIBC-AuNPs (**d**).

To study the molecule chirality effect on amylin amyloidosis at the nano-bio interface, the fibrillation kinetics of amylin in presence of *L*- or *D*-NIBC-AuNPs were evaluated by a standard ThT binding assays (Figure 2a,b). Meanwhile, the concentration of amylin was first adjusted through the preliminary experiment (Figure S3 in the Supplementary Materials) and then maintained 10 μmol·L^−1^ in the following experiments. It is well known that the fibrillation process of amylin can be mainly divided into nucleation and elongation phases [40]. In this experiment, *L*- or *D*-NIBC-AuNPs with different concentrations of 1, 5, 20, 50 mg·L^−1^ were added to the amylin solution (10 μmol·L^−1^) at the beginning of incubation at 37 °C. Without chiral additives (black curves in Figure 2a,b), amylin began to aggregate at the 3rd hour and completely fiberized after the 8th hour, which showed typical sigmoidal growth curves with three growth stages, including an initial lag period, a fast elongation phase and a final plateau phase [41]. All curves were normalized by taking the fluorescence intensity of the plateau phase in the growth curves of the pure amylin as unit 1. As shown in Figure 2a,b, the fluorescence intensities of the plateau phase in the corresponding growth curves declined to a different degree with the increasing dosage of both two chiral AuNPs. For the sample with the addition of *D*-NIBC-AuNPs (Figure 2b), 1, 5, 20 and 50 mg·L^−1^ of them reduced the ThT fluorescence intensities to 96%, 50%, 40% and 30%, respectively. Whereas the same addition of *L*-NIBC-AuNPs reduced the ThT fluorescence intensities to 85%, 44%, 29% and 10% (Figure 2a), respectively. As known to all, the fluorescence intensity was proportional to the total amount of amylin fibers [42]. Another control experiment showed that free NIBC enantiomers did not have an evidential influence on amylin fibrillation under the same conditions (Figure S4 in the Supplementary Materials). The above results demonstrated that both two chiral AuNPs could inhibit the fiber elongation process of amylin in a dosage-dependent manner, while the *L*-NIBC-AuNPs provided higher inhibition efficiency than *D*-NIBC-AuNPs. On the other hand, the addition of *D*-NIBC-AuNPs (Figure 2b) brought forward the nucleation phase in a dosage-dependent manner, while *L*-NIBC-AuNPs, remarkably, postponed this process for about 2 h without dosage discrimination. This result directly indicated that the chirality of AuNPs ligands greatly influenced the nucleation of amylin. All the above results demonstrated that the molecule chirality at the nano-bio interface plays a key role in both the nucleation and the fiber elongation of proteins/peptides amyloidosis.

In order to confirm this speculation, AFM was used to represent topographic characteristics of amylin aggregates after co-incubated amylin in the absence/presence of *L*(*D*)-NIBC-AuNPs with different concentrations for 24 h. As shown in Figure 2c,d, increasing the dosage of both two chiral AuNPs remarkably reduced the amount of amylin fibrils and *L*-NIBC-AuNPs showed better inhibitory efficiency than the same dosage of *D*-NIBC-AuNPs. This result was consistent with the ThT kinetics result. Interestingly, for the samples with the addition of *L*-NIBC-AuNPs, amorphous aggregates increased with the additive dosage. That differed from the samples with the addition of *D*-NIBC-AuNPs, in which the length and number of amylin fibrils decreased with the additive dosage. This result not only further proved that the chirality of ligands at the nano-bio interface determined the inhibitory efficiency but also revealed a different inhibition mechanism induced by molecule chirality at the nano-bio interface.

### 3.3. The Similarities of L-and D-NIBC-AuNPs in Inhibition Mechanisms

**Figure 3:**
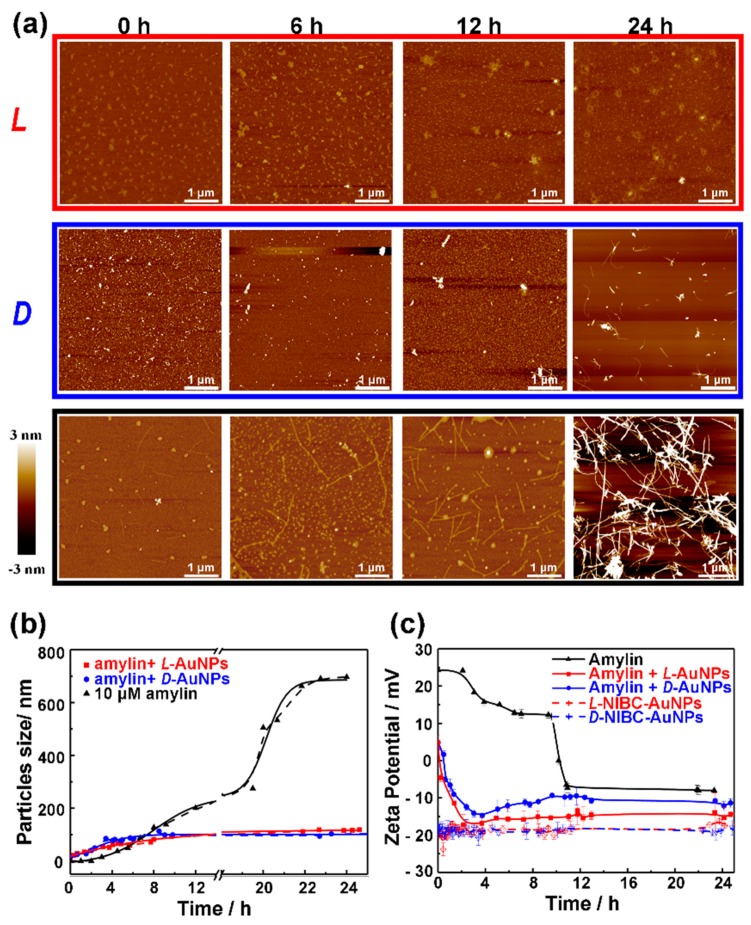
(**a**) Atomic force microscopy (AFM) images of amylin (10 μmol·L^−1^) co-incubated without (black border) or with 20 mg·L^−1^ *L*(*D*)-NIBC-AuNPs (red border: *L*-NIBC-AuNPs, blue border: *D*-NIBC-AuNPs) in PBS at 37 °C (Samples collected at 0, 6th, 12th and 24th h of the incubation). (**b**,**c**) Time-dependent average sizes (**b**) and Zeta potential changes (**c**) of amylin (10 μmol·L^−1^) co-incubated without (black curve)/with 20 mg·L^−1^ *L*(*D*)-NIBC-AuNPs (red: *L*-NIBC-AuNPs, blue: *D*-NIBC-AuNPs) in ultrapure water at 37 °C; dotted curves in c: pure *L*(*D*)-NIBC-AuNPs.

To explore the inhibitory mechanisms, we further tracked the fibrillation process of amylin in the absence or presence of chiral AuNPs by AFM and the samples were collected at 0, 6th, 12th and 24th h. As shown in Figure 3a, the amylin aggregated into mature fibrils (black frame in Figure 3a) gradually over time, while no mature fibrils could be found when co-incubated amylin with 20 mg·L^−1^ *L*-NIBC-AuNPs (red frame in Figure 3a) or *D*-NIBC-AuNPs (blue frame in Figure 3a) even after 24 h. Considering a similar inhibitory effect has also been observed for other ligands coated AuNPs in our previous work [26], we concluded that the inhibitory effect was mainly attributed to the size of AuNPs, not confined to a specific ligand. The physical and chemical properties, including the chirality of ligands at the nano-bio interface can only regulate the inhibitory efficiency. Notably, the amorphous structures or short rod-like aggregates which were in accordance with AFM studies in Figure 2c began to appear at the 6th hour in the corresponding samples with addition of *L*- or *D*-NIBC-AuNPs.

Time-dependent average sizes (Figure 3b) and zeta potential (Figure 3c) of 10 μM amylin co-incubated without/with 20 mg·L^−1^ *L*(*D*)-NIBC-AuNPs in ultrapure water were recorded in cuvettes by using the in-situ real time measurement. Notably, in order to eliminate the interference of salt ions to the detection of sizes and zeta, we intentionally employed ultrapure water instead of PBS as the solvent. As shown in Figure 3b, the apparent sizes of amylin aggregates increased gradually from 1.9 nm to about 87.1 nm at the initial incubation period of 6 h, after which their sizes increased sharply to 696.5 nm within 18 h. These stages delayed behind to the lag phase (i.e., the oligomer formation and nucleation process) and growth phase (i.e., fibril formation process) observed in ThT assay (Figure 2a,b) of amylin fibrillation. That could be due to the lack of PBS in the sample solution. Interestingly, when co-incubated with chiral AuNPs, the sizes were a litter larger than the amylin alone at each time point within the initial 6 h, after which their sizes remained almost unchanged during the following incubation period. No significant difference could be found between two chiral samples. This result further proved that the inhibitory effect was mainly attributed to the size of the AuNPs, not confined to a specific ligand.

### 3.4. The Differences of L-and D-NIBC-AuNPs in Inhibition Mechanisms

To better understand the inhibitory mechanisms of *L*(*D*)-NIBC-AuNPs in the amylin fibrillation process, the profiles of time-dependent zeta potential are shown in Figure 3c. The zeta potential of monomeric amylin in ultrapure water was +24.3 mV at the beginning of incubation. With incubation time prolonging, the surface charge of the amylin aggregates gradually decreased to +12.5 mV within the initial incubation period of 9 h. This could be explained by some positive charge residues of amylin were locked in the interior of aggregates during the monomers self-assemble into oligomers or short fibrils. Then the surface zeta potential of amylin aggregates decreased sharply from +12.5 to −7.3 mV in the following 3 h (from 9th to 12th h). This is because more positive charge residues of amylin were locked in the interior of aggregates with the negative charge residues remained outside during the formation of mature fibrils. On the other side, both *L*-NIBC-AuNPs and *D*-NIBC-AuNPs were negatively charged in the ultrapure water with a zeta potential at about −20 mV. When 10 μmol·L^−1^ amylin co-incubated with 20 mg·L^−1^ *L*(*D*)-NIBC-AuNPs, amylin monomers or oligomers would be absorbed rapidly onto the surface of both two chiral AuNPs via electrostatic interaction, leading to the initial zeta potential of both two mixtures appearing at +4.9 mV. More interestingly, the zeta potential of mixtures reduced quickly in the initial 2.5 h from +4.9 mV to −17.2 or −14.1 mV for the addition of *L*-NIBC-AuNPs or *D*-NIBC-AuNPs, respectively. Then, the zeta potential of both two mixtures remained unchanged throughout the incubation period. The zeta potential of amylin co-incubated with *L*-NIBC-AuNPs for 24 h was lower than that of *D*-NIBC-AuNPs. It may be attributed to the different structures formed in the corresponding samples. The amount of *L*-NIBC-AuNPs binding onto the amorphous structures were more than the *D*-NIBC-AuNPs binding onto the short rod-like aggregates (Figure 3). These results further proved that molecule chirality played a key role in the stereoselective interaction between nano-bio interface and protein/peptides.

**Figure 4:**
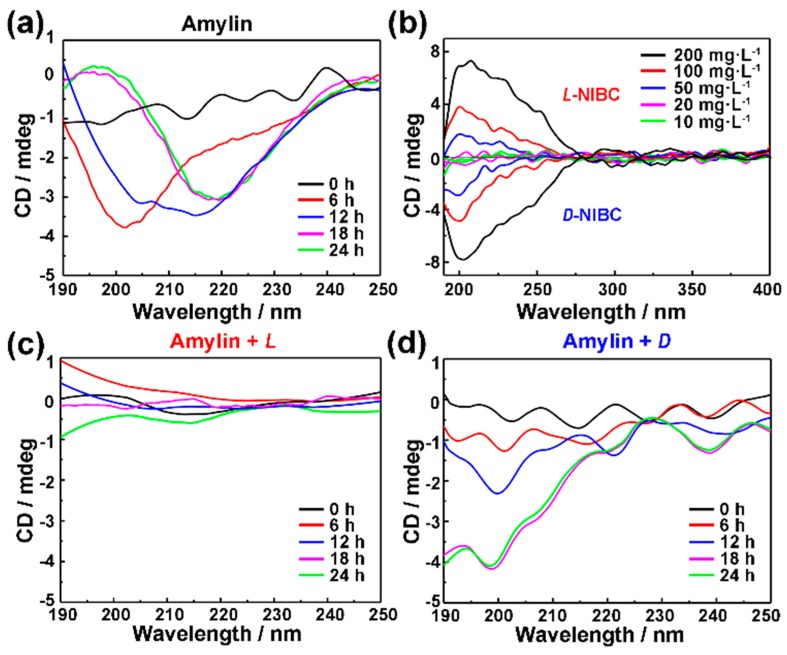
Time-dependent CD spectra profiles of 10 μM amylin co-incubated without (**a**) with 20 mg·L^−1^ *L*-NIBC-AuNPs (**c**) and *D*-NIBC-AuNPs (**d**) in ultrapure water at 37 °C. The colors of the curves represented different time points of incubation period, black: 0, red: 6th h, blue: 12th h, purple: 18th and green: 24th h. (**b**) The CD spectra of *L*(*D*)-NIBC-AuNPs with different concentration.

Furthermore, in-situ real time CD spectra were employed to monitor the secondary structures conversion of amylin co-incubated with/without *L*(*D*)-NIBC-AuNPs over a period of time during the aggregation progress. As shown in Figure 4a, 10 μmol·L^−1^ amylin experienced a typical conformational transition, which was a prerequisite for the aggregation and fibrillation of amylin during the whole incubation period. The random coils (no characteristic signal) transformed into α-helix (single characteristic negative peak at 203 nm) in the initial 6 h. Then the α-helix transformed into β-sheet (negative peak at 218 nm) within the following 12 h. These two conformational transitions corresponded to the two growth stages (nucleation and elongation) in the fibrillation of amylin [43]. In order to eliminate the signal interference of the chiral AuNPs themselves on the amylin aggregates, the CD spectra of *L*(*D*)-NIBC-AuNPs with different concentrations had been checked (Figure 4b). The intensity of CD signal decreased with the decreasing concentration of chiral AuNPs. Notably, 20 mg·L^−1^ chiral AuNPs showed no CD signal at the wavelength range of 190–250 nm. This meant that the 20 mg·L^−1^ chiral AuNPs had no signal interference on the amylin aggregates. When 10 μmol·L^−1^ amylin co-incubated with 20 mg·L^−1^ *L*-NIBC-AuNPs (Figure 4c), there was no characteristic signal could be found even after incubation for 24 h. This result implied that the *L*-NIBC-AuNPs inhibited the random coils of amylin transformed into α-helix in the nucleation stage. On the other hand, though the addition of *D*-NIBC-AuNPs could not stop the random coils of amylin transformed into α-helix in the nucleation stage, they could postpone this process (Figure 4d). Moreover, the CD signal showed no change in the last 6 h (from 18th to 24th h), which demonstrated that the addition of *D*-NIBC-AuNPs could inhibit the α-helix transformed into β-sheet in the elongation stage. In addition, the same amount of *L*(*D*)-NIBC had no effect on the conformational transition of amylin (Figure S5 in the Supplementary Materials). These results indicated that the molecule chirality at the nano-bio interface greatly influenced the initial conformational transition of amylin.

### 3.5. Cytotoxicity Tests

**Figure 5:**
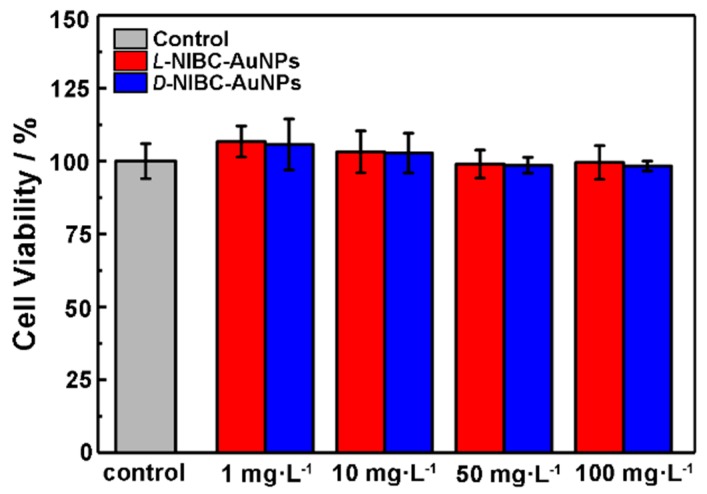
Viabilities of INS-1 cells after co-incubated with *L*-NIBC-AuNPs (red) or *D*-NIBC-AuNPs for 24 h with different concentrations from 1 to 100 mg·L^−1^, respectively. Data represented as mean ± SD (*n* = 3).

The above results showed that both two chiral AuNPs have the capacity to inhibit the amylin amyloidosis in vitro. In order to preliminarily investigate whether the chiral AuNPs could be applied in vivo in the future, the cytotoxicity of chiral AuNPs were tested by using INS-1 cells. The viabilities of INS-1 cells with or without additions of different concentrations of *L*(*D*)-NIBC-AuNPs were examined by a standard CCK-8 fluorescence assay. The results were presented in Figure 5, both two chiral AuNPs did not exhibit obvious toxicity within a wide concentration range of 1–100 mg·L^−1^ to INS-1 cells. In addition, the cytotoxicity test of the chiral ligands demonstrated that both the *L*(*D*)-NIBC enantiomers were non-cytotoxicity even at a high-dosage of 100 mg·L^−1^ (Figure S6 in the Supplementary Materials). These results indicated that the chiral molecule modified AuNPs had a good biocompatibility, which had a promising potential for in vivo applications.

## 4. Conclusions

In this work, we built a chiral nano-bio surface, taking *L*(*D*)-NIBC-AuNPs as a model, to study the chiral effect on the fibrillation of amylin. Both chiral AuNPs could inhibit amylin aggregation in a dosage-dependent manner. The time-dependent size-changing of amylin co-incubated with *L*(*D*)-NIBC-AuNPs showed no obvious increase in size. The time-dependent zeta-changing and the in-situ real time CD spectra explained the different mechanisms between two chiral AuNPs, which was that *L*-NIBC-AuNPs could inhibit the conformation transition process of amylin from random coils to α-helix, while *D*-NIBC-AuNPs could only delay but not prevent the formation of α-helix, however they could inhibit the further conformation transition process of amylin from α-helix to β-sheet. Besides, both chiral AuNPs had an inhibitory effect on the nucleation period. This work can not only help us understand how molecule chirality at a nano-bio interface works in vivo but also provides a strategy for designing an inhibitor for anti-amyloidosis targeting diverse neurodegenerative diseases.

## Supplementary Materials

The following are available online at https://www.mdpi.com/2079-4991/9/3/412/s1, Figure S1: TEM images of *L*(*D*)-NIBC-AuNPs, Figure S2: XPS spectra of *L*(*D*)-NIBC-AuNPs, Figure S3: Time-dependent ThT fluorescence profiles of amylin incubation with different concentrations and AFM image of amylin fibrils, Figure S4: Time-dependent ThT fluorescence profiles of amylin co-incubated in the absence or presence of *L*(*D*)-NIBC with different concentrations, Figure S5: Time-dependent CD spectra profiles of amylin co-incubated with *L*(*D*)-NIBC, Figure S6: Cytotoxicity tests of *L*(*D*)-NIBC with different concentrations.
