# Supplementary material for: Chiral Effect at Nano-Bio Interface: A Model of Chiral Gold Nanoparticle on Amylin Fibrillation
Source: Nanomaterials (Basel). 2019 Mar 11;9(3):412. doi: 10.3390/nano9030412 (PMC6474006; doi:10.3390/nano9030412)
Supplement: Supplementary file 1 [file nanomaterials-09-00412-s001.zip › nanomaterials-449526-SI.pdf]

Supporting Information

Chiral Effect at Nano-Bio Interface: A Model of Chiral Gold Nanoparticle on Amylin Fibrillation

Jing Li [1,†], Rui Chen[1,†], Shasha Zhang[2,†], Zhongjie Ma[1], Zhuoying Luo[1] and Guanbin Gao[1,*]

[1] State Key Laboratory of Advanced Technology for Materials Synthesis and Processing, Wuhan University of Technology, No.122 Luoshi Road, Wuhan 430070, China;
[2] School of Arts and Media, Wuhan Vocational College of Software and Engineering, No. 117 Guanggu Road, Wuhan 430205, People's Republic of China;
[†] These authors contributed equally.
* Correspondence: gbgao@whut.edu.cn, +86-027-8765-1837.

Content

1. **Figures and relevant discussion**

1.1 TEM images of *L*(*D*)-NIBC-AuNPs. (Fig. S1)

1.2 XPS spectra of *L*(*D*)-NIBC-AuNPs. (Fig. S2)

1.3 Time-dependent ThT fluorescence profiles of amylin incubation with different concentrations and AFM image of amylin fibrils. (Fig. S3)

1.4 Time-dependent ThT fluorescence profiles of amylin co-incubated in the absence or presence of *L*(*D*)-NIBC with different concentrations. (Fig. S4)

1.5 Time-dependent CD spectra profiles of amylin co-incubated with *L*(*D*)-NIBC. (Fig. S5)

1.6 Cytotoxicity tests of *L*(*D*)-NIBC with different concentrations. (Fig. S6)

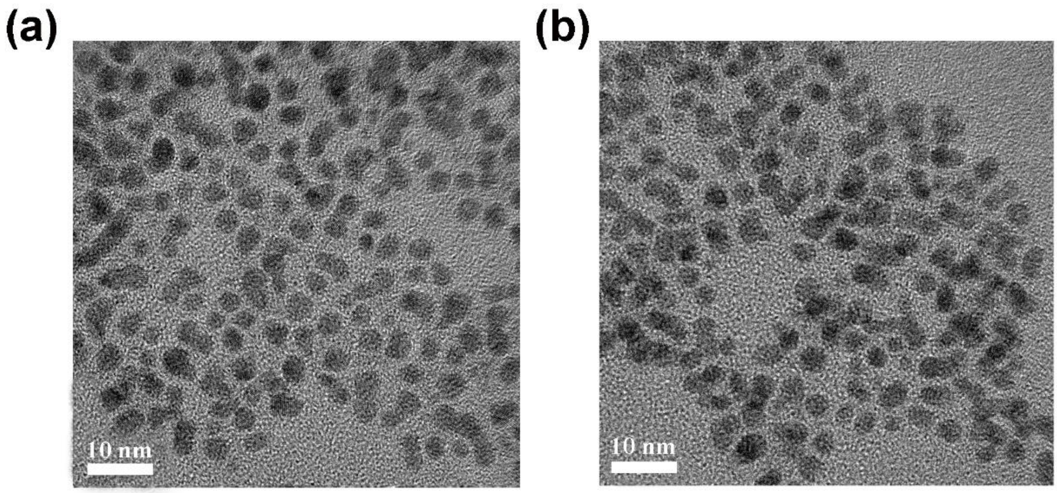

Figure S1 HR-TEM images of *L*-NIBC-AuNPs (a) and *D*-NIBC-AuNPs (b).

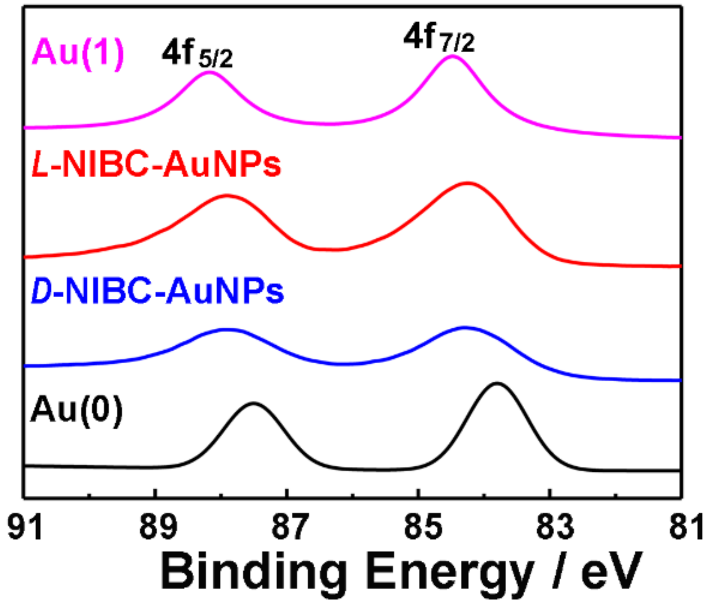

Figure S2 XPS spectra for *L*(*D*)-NIBC-AuNPs. Black: *L*-NIBC-AuNPs, red: *D*-NIBC-AuNPs.

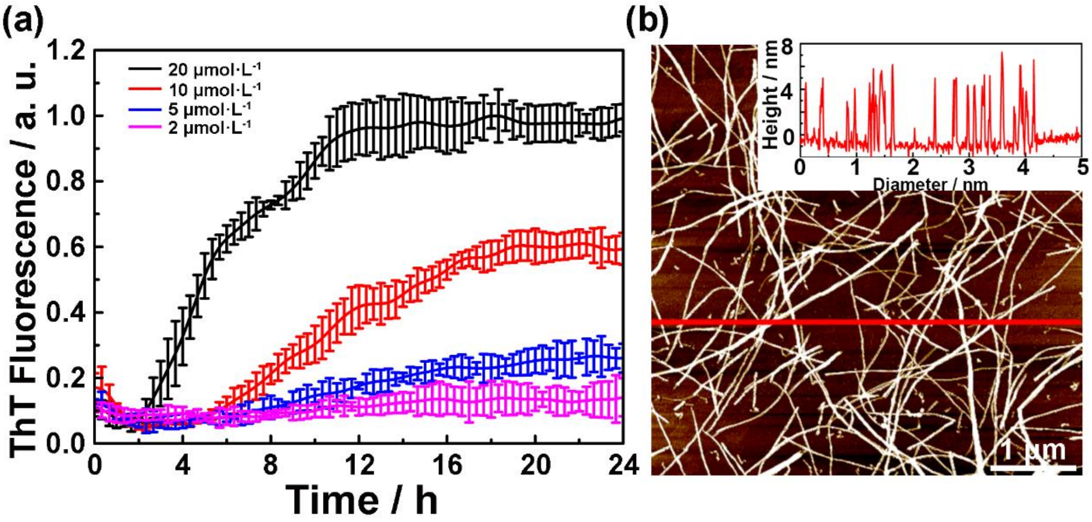

Figure S3 (a) Time-dependent ThT fluorescence profiles of amylin incubation with different concentrations at 37 °C. The colors of the curves represented different concentrations of amylin, black: 20 μmol·L^{-1}, red: 10 μmol·L^{-1}, blue: 5 μmol·L^{-1}, purple: 2 μmol·L^{-1}; each curve displayed the average data of three parallel experiments. Data points and error bars shown represent mean ± SE, (n=3). (b) AFM image of amylin fibrils after 10 μmol·L^{-1} amylin incubated alone for 24 h at 37 °C.

Figure S3a showed the fibrillation kinetics of amylin with different concentrations. The fluorescence intensity increased with the increasing of amylin concentration, and the typical sigmoidal growth curves with three growth stages, including an initial lag period, fast elongation phase and final plateau phase, could be observed when the concentration of amylin reached 10 μmol·L^{-1}. So the amylin concentration of 10 μmol·L^{-1} was used in the following experiments. On the other hand, a large number of mature fibrils (Figure S3b) could be found in the sample of 10 μmol·L^{-1} amylin incubation alone for 24 h, which clearly indicated that amylin could self-assemble into mature fibrils at concentration of 10 μmol·L^{-1}.

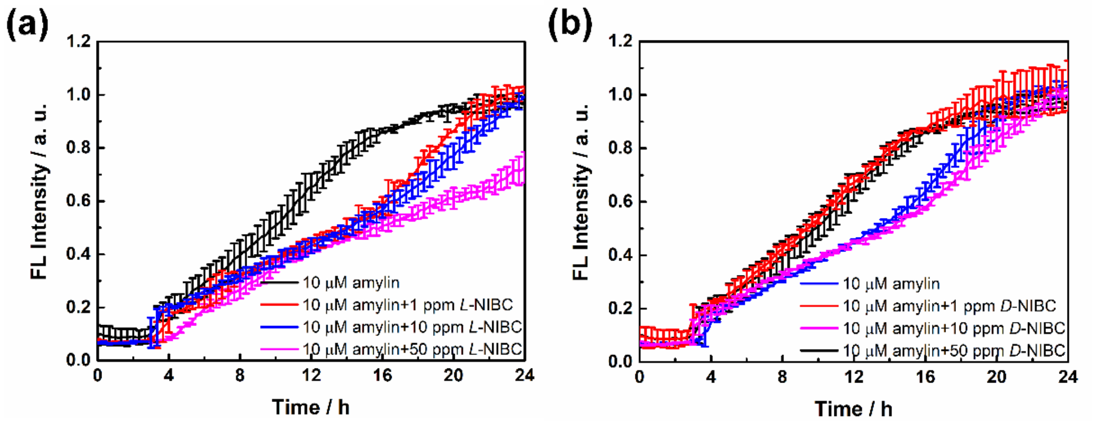

Figure S4 Time-dependent ThT fluorescence profiles of 10 μmol·L^{-1} amylin co-incubated in the absence (control, black) or presence of *L*-NIBC (a) and *D*-NIBC (b) with different concentrations at 37 °C. The colors of the curves represented different concentrations of *L*-/*D*-NIBC, red: 50 mg·L^{-1}, blue: 10 mg·L^{-1}, purple: 1 mg·L^{-1}; each curve displayed the average data of three parallel experiments. Data points and error bars shown represent mean ± SE, (n=3).

Figure S4 showed that there was no obvious difference between amylin alone and addition of *L*-/*D*-NIBC, which indicated *L*-/*D*-NIBC could not inhibit amylin fibrillation progress. When compared to *L*-/*D*-NIBC AuNPs, taking 5 mg·L^{-1} *L*-NIBC-AuNPs for example, it contained 1.53 mg·L^{-1} *L*-NIBC formed Au-S bond. All above evidences have proved that it was exactly gold nanoparticles that play an important role in arrest amylin fibrillation rather than *L*-/*D*-NIBC alone.

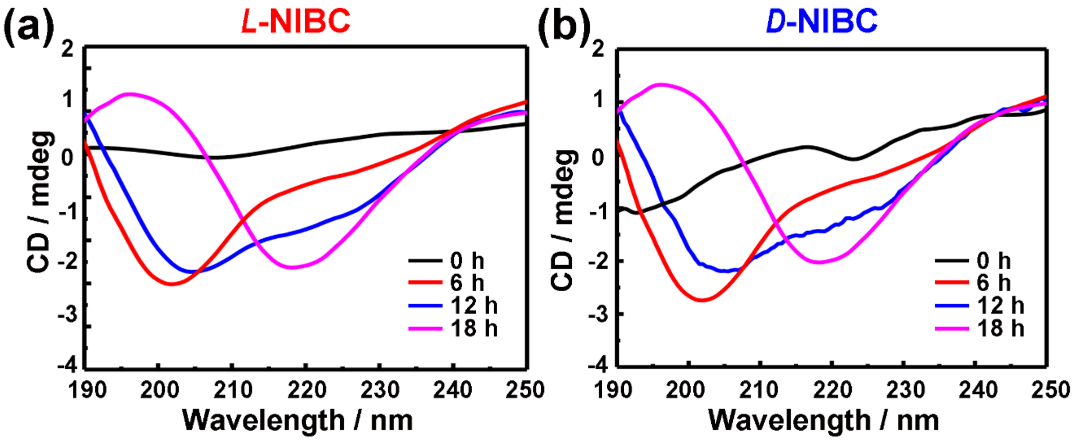

Figure S5 Time-dependent CD spectra profiles of 10 μmol·L^{-1} amylin co-incubated with 6.2 mg·L^{-1} *L*-NIBC (a) and *D*-NIBC (b).

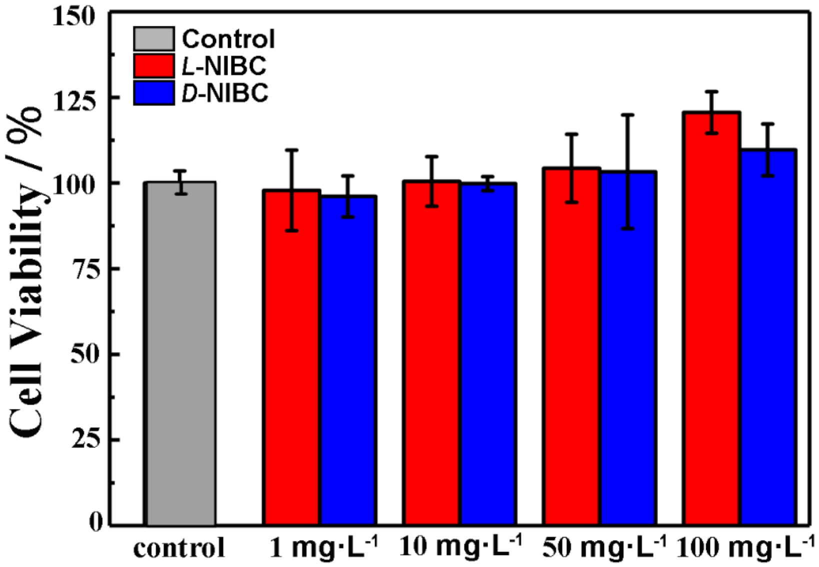

Figure S6 Cytotoxicity tests of *L*(*D*)-NIBC with different concentrations from 1 to 100 mg·L^{-1} for 24 h, respectively. Data represented as mean ± SD (n = 3).
